# Exploring the Potential of Indigenous Foods to Address Hidden Hunger: Nutritive Value of Indigenous Foods of Santhal Tribal Community of Jharkhand, India

**DOI:** 10.1080/19320248.2016.1157545

**Published:** 2016-05-05

**Authors:** Suparna Ghosh-Jerath, Archna Singh, Melina S. Magsumbol, Preeti Kamboj, Gail Goldberg

**Affiliations:** ^a^ Indian Institute of Public Health–Delhi, Public Health Foundation of India, Gurgaon, India; ^b^ All India Institute of Medical Sciences, Ansari Nagar, New Delhi, India; ^c^ Public Health Foundation of India, New Delhi, India; ^d^ Nutrition and Bone Health Research Group, MRC Human Nutrition Research, Elsie Widdowson Laboratory, Cambridge, UK

**Keywords:** Indigenous foods, santhal tribes, micronutrients, hidden hunger

## Abstract

Traditional foods of indigenous communities can be explored as a sustainable means of addressing undernutrition. Our study aimed at identifying indigenous foods of the Santhal tribal community of Godda district of Jharkhand, India, assessing their nutritive value, and appraising their potential role in addressing hidden hunger. A cross-sectional survey using qualitative methods like focus group discussions with women of childbearing age (15–49 years), adult males, and elderly people was conducted for food identification. This was followed by taxonomic classification and quantitative estimate of nutritive value of the identified foods either in a certified laboratory or from secondary data. The community was well aware of the indigenous food resources in their environment. More than 100 different types of indigenous foods including a number of green leafy vegetables were identified. Taxonomic classification was available for 25 food items and an additional 26 food items were sent for taxonomic classification. Many indigenous foods (more than 50% of which were green leafy vegetables) were found to be rich sources of micronutrients like calcium, iron, vitamin A as beta carotene, and folate. Maximizing utilization of indigenous foods can be an important and sustainable dietary diversification strategy for addressing hidden hunger in this indigenous community.

## Background

Indigenous people are those who retain knowledge of the land and food resources rooted in historical continuity within their region of residence. The food systems of indigenous people often include “traditional foods”; that is, those that are not purchased but obtained locally from the natural environment. They are chiefly procured either through farming or wild harvesting and utilized based on traditional wisdom and knowledge.^1^ It is well recognized that traditional foods and dietary diversity within an ecosystem can be powerful sources of nutrients and thus are better for health.^2^ Various ethnobotanical surveys indicate that several species of wild plants have been used for human food at some stage of human history.^3^,^4^ Use of several species of plants, wild fungi, and edible insects has also been documented.^5^ ^–^ ^8^ Bushmeat and fish are reported as providing 20% of protein in many developing countries^8^ and indigenous foods in the Gambia have been shown to be important sources of calcium.^9^ The diversity in wild species augments the variety of family diets and may contribute to household food security.^10^ However, the health benefits of many of these indigenous foods have been largely unexplored and research on the nutritive value of underutilized species/local varieties deserves a higher priority in nutrition research.

Dietary diversification is a proven cost-effective strategy to ameliorate malnutrition. The loss of dietary diversity has many implications for the nutrition and health of rural communities including loss of income generation and decreased consumption of diverse foods. The multiple benefits of preservation and promotion of indigenous foods range from a collateral benefit on biodiversity and environmental sustainability to improving micronutrient intakes.^11^

The tribal communities in India are a good example of indigenous populations with a vast diversity in their cultures, traditions, and environments. The numerous indigenous foods that exist in the Indian tribal environment reflect the rich biodiversity of India that can be potentially used to promote food security, nutrition, and health. Some of these indigenous foods have been analyzed and documented from different regions across India.^2^,^5^,^12^ ^–^ ^15^ However, a comprehensive nationwide database is still to evolve and many indigenous foods are yet to be listed and their nutritive value analyzed.

In addition, despite this wealth of traditional knowledge of natural resources in these tribal communities, challenges of geography, agricultural technology, cultural habits, lack of formal education, poor infrastructure, and poverty may contribute to poor nutrition and health.^2^ A high prevalence of chronic energy deficiency and undernutrition along with micronutrient deficiency among tribal populations is well documented.^16^ ^–^ ^18^ Children belonging to tribal communities are at higher risk of iron-deficiency anaemia and vitamin A deficiency disorders. In addition, in women of certain rural and tribal communities, zinc, vitamin B12, and iron constitute the principal micronutrient deficiencies.^19^,^20^ Chronic micronutrient deficiency resulting from insufficient intake of vitamins and minerals is often referred to as “hidden hunger.” It results from lack of dietary diversity and suboptimal and poor-quality food intake and affects around 2 billion people worldwide.^21^ As the name indicates, the signs and symptoms of undernutrition and hidden hunger are less overtly visible in those affected by it^22^ compared to the immediate response to lack of adequate food; that is, hunger.

The state of Jharkhand in India is among the states and union territories with a significant tribal population. Jharkhand has a total of 30 Scheduled Tribes (an indigenous group of people officially regarded as socially disadvantaged in India). Out of these, Santhal is the most populous tribal community.^23^ Studies have reported a high prevalence of undernutrition, chronic energy deficiency, and iron deficiency in the adults and children of the Santhal community residing in different states of India, including Jharkhand, Orissa, and West Bengal.^24^ ^–^ ^28^ Studies have also documented a wide variety of indigenous foods that are consumed by this community.^15^ However, documentation of the nutritive value of many of these foods is not available.

The present study was undertaken to explore the range of indigenous foods consumed by Santhal tribal community of Jharkhand, India. The specific focus was on analyzing their nutritive values and to appraise the potential of these foods in addressing micronutrient deficiencies.

The study involved listing, identification, and taxonomic classification of indigenous foods, followed by nutrient composition analysis, if their nutritive values were not found to be documented in the Indian Food Composition tables.^29^

## Materials and methods

This was an exploratory cross-sectional study conducted in 4 selected villages inhabited by Santhal tribal community in Godda district of Jharkhand, India. The 4 villages were identified using probability proportional to size sampling^30^ based on a verified list of villages inhabited by Santhal community.

The data collection was conducted between March 2013 and November 2013; multiple visits were scheduled to capture the diversity of foods that were consumed during each season. In addition to the core research team, the study team also included well-trained non-governmental organization (NGO) workers fluent in the native Santhal dialect.

### Study procedures

#### Participatory rapid assessment

Participatory rapid assessment (PRA) methods were used to elicit information on commonly consumed local foods. Focus group discussions (FGDs) were conducted to assess the range of available foods and the contribution of indigenous wild foods to the regular diets of the Santhal community. The female community health workers or *sahiyyas* in the respective villages were requested to invite community members to participate in the FGDs ahead of the field visits. The participants included women with children, adult men, and the elderly (men and women). Mothers were especially encouraged to attend because they were mainly responsible for food preparation and feeding their families. The FGDs were held in accessible areas such as the *Anganwadi* centers (community centers for children) or in front of the homes of the *sahiyyas*. During the FGDs, a discussion guide was used by the study team to steer the conversation toward the participants’ knowledge of food groups, foraging and hunting activities, and rearing of animals for food. The study team with the help of the local NGO workers explained the nature of the study and obtained signed written consents from literate participants. Those who could not read or write gave verbal consents with a third-party signatory. Copies of the translated study information sheets and consent forms were given to participants. All participants were informed that the FGDs were going to be recorded and that no personal information would be used in any of the study reports. Permission was taken for pictures to be taken during the FGDs. The local NGO workers transcribed and translated the discussions to Hindi or English. Personal or identifiable information was not recorded in any reports. The aforementioned description of the study thus adhered to the RATS guidelines for reporting qualitative studies.

During the PRA exercise the following methods were adopted:

1. The FGD included a free listing exercise to identify indigenous foods consumed in the community and develop a list of such food items. The participants were then asked to identify indigenous or *deshi* foods gathered from the local environment such as nearby forests (jungle), fields, agricultural fields, gardens (*bari* or kitchen garden), or water resources such as man-made ponds (*pokhar*), creeks, or dams or even those bought from the weekly markets (*haat*). The local names of plants or meat items and their characteristics such as availability, seasonality, and source were documented. The foods identified were then categorized under various food groups based on their edible parts. Ethnographic manuscripts on the tribal populations in the area (formerly Bihar state) were also used to confirm the list of common foods.^31^ The text of the FGDs was reviewed through thematic analysis. Atlas.ti version 7^32^ was used by 2 researchers who independently coded and analyzed the content of the transcripts. Codes were created to help identify overarching themes or similar items under each theme.
2. Pairwise ranking: Pairwise ranking was used to identify perceptions, priority setting, and preferences for local food items. After the free listing exercise, the FGD participants were asked to identify 5 to 6 preferred food items within each food group; for example, green leafy vegetables (GLVs), cereals, vegetables, etc. These preferences were based on criteria of taste and availability of particular food items. These were then ranked. This helped in identifying the popular and commonly consumed indigenous foods under different food groups.^33^

The most commonly consumed food items as identified under each food group category were then entered into a matrix on a flip chart. An example of pairwise ranking is provided in Table 1. Participants were then asked to compare the first food item in the row with various food items listed in the column one by one. The next step was to ask them to move on to the second food item in the row, keeping that as a constant, and comparing it with the third and the subsequent food items and enter the preference in the relevant grid. The same steps were repeated until all of the food items listed in the row were compared with the subsequent food items listed in the columns pairwise. A score was provided based on the number of times each food item was selected.^32^ Using this method, a hierarchy of preferred food items in the various food groups was identified.

**Table 1.** Pairwise ranking of traditional rice varieties.^a^

	Jadhan	Swarna	Lohna	Bhadai	Jondra
Jadhan	×	Jadhan	Jadhan	Bhadai	Jadhan
Swarna	Jadhan	×	Lohna	Swarna	Swarna
Lohna	Jadhan	Lohna	×	Bhadai	Lohna
Bhadai	Bhadai	Swarna	Bhadai	×	Bhadai
Jondra	Jadhan	Swarna	Lohna	Bhadai	×

^a^Scores: Jadhan, 6; Swarna, 4; Lohna, 3; Bhadai, 6; Jondra, 0. The participants preferred jadhan and bhadai among the identified traditional varieties of rice.

#### Identification of food samples

Based on the free listing activity done through FGDs, a list of commonly consumed indigenous food items was compiled (including cereals, roots and tubers, legumes/pulses, vegetables, GLVs, seeds, fruits, and animal foods). A literature search was done to identify the taxonomic classification based on the common names provided by the community. Samples were collected for those food items whose taxonomic names were not found, and these were sent for classification to an expert team at the Department of Botany, Birsa Agricultural University, Ranchi, Jharkhand.

#### Procedure for sample collection for classification

One sample of the food (around 50–100 g) was collected from the field, wrapped in paper towels, and put in a well-perforated polythene bag and sent to the team of experts for identification of taxonomic classification. The food items being sent for classification were photographed and added to the documentation inventory.

After identification and subsequent verification regarding the availability of the nutritive value of the identified food in the Indian food composition tables, a list of food items was prepared for the purpose of sample collection for nutrient analysis.

#### Collection of food samples for analysis

The food samples short listed for nutrient analysis were collected from the field site or procured from the local market (whichever was the usual mode of procurement in the community). Each of the samples was dusted to remove excess soil/dirt taking care to avoid mechanical damage and air dried to remove extraneous moisture. The samples were then weighed, wrapped in clean paper towels, placed in well-perforated polythene bags, and placed in a carrier lined with ice packs before being transported to the site of storage and analysis by train. Five hundred grams of each of the vegetables/fruits/green leafy vegetables/tubers and 500 ml of indigenous alcohol samples were sent to the National Accreditation Board for Testing and Calibration Laboratories (NABL) certified laboratory for analysis.

#### Nutrient analysis

Nutrient analysis was done according to standard reference protocols (Table 2). The analyte values were reported per 100 g of edible weight. All analyses were performed in duplicates. The raw/uncooked samples were analysed for parameters including energy (Codex Guidelines for Nutritional Labelling _CAC/GL 2-1985),^34^ protein (IS 7219- 1973),^35^ total fat (IS-4684-1975),^36^ total carbohydrates (by calculation),^37^ sugar (titration; FSSAI manual of methods),^38^ and dietary fiber (AOAC 991.43).^39^ The vitamins including vitamin A (as beta carotene)40, thiamine (vitamin B1),^41^ riboflavin (vitamin B2),^41^ niacin (Vitamin B3),^41^ were estimated by HPLC based UV-visual detection, vitamin C (as L-ascorbic acid)^42^ by titration (IS 5838-1970) and folates (as folic acid) by the BioRad ELISA kit, MA USA. The minerals i.e. calcium, iron, zinc, sodium were analyzed based on the AOAC 999.10 methodology.^43^ Three local alcoholic beverages were also analyzed for total ethanol content in addition to the other nutrients. The laboratory followed standard quality control and quality assurance programs (including participation in proficiency testing programs) as part of the analytical methodology.

**Table 2.** List of parameters and relevant specifications for nutrient analysis.^a^

S. No.	Test parameter	Unit	Reported method of testing	Reference method of testing	Instrument used (quality control checks done for all instruments as prescribed)
1	Energy	kcal/100 g	IFS/C/STP/FC/008	Codex Guidelines for Nutritional Labelling _CAC/GL 2-1985	By calculation
2	Protein (*N* × 6.25)	%	IS 7219-1973	IS 7219-1973	Kjeldahl digestion apparatus
3	Total fat	%	IFS/C/STP/FC/012	IS-4684-1975 Reaffirmed 1983	Soxhlet apparatus
4	Total carbohydrate	%	IFS/C/STP/FC/013	AOAC 986.25	By calculation
5	Sugar	%	IFS/C/STP/FC/010	FSSAI Manual of methods of Analysis of Food, Lab Manual 4 (32)	Titration
6	Dietary fiber	%	IFS/C/STP/FC/007	AOAC 991.43	Gravimetric
7	Vitamin A (as beta carotene)	mg/100 g	IFS/C/STP/LC/025	International Food Research Journal 19(2): 531-535 (2012)	Thermofisher Scientific HPLC UV-Vis with C-18 column
8	Vitamin B_1_	mg/100 g	IFS/C/STP/LC/002	Food analysis by HPLC (33)	Thermofisher Scientific HPLC UV-Vis with C-18 column
9	Vitamin B_2_	mg/100 g	IFS/C/STP/LC/002	Food analysis by HPLC (33)	Thermofisher Scientific HPLC UV-Vis with C-18 column
10	Vitamin B_3_	mg/100 g	IFS/C/STP/LC/002	Food analysis by HPLC (33)	Thermofisher Scientific HPLC UV-Vis with C-18 column
11	Vitamin C	mg/100 g	IS 5838-1970	IS 5838-1970	Titration
12	Calcium	mg/100 g	IFS/C/STP/AAS/004	AOAC 999.10	Thermofisher Scientific AAS
13	Iron	mg/100 g	IFS/C/STP/AAS/004	AOAC 999.10	Thermofisher Scientific AAS
14	Zinc	mg/100 g	IFS/C/STP/AAS/004	AOAC 999.10	Thermofisher Scientific AAS
15	Sodium	mg/100 g	IFS/C/STP/AAS/004	AOAC 999.10	Thermofisher Scientific AAS
16	Folic acid	µg/kg	IFS/M/STP/027	BioRad ELISA Kit	ELISA reader

HPLC indicates high-performance liquid chromatography; UV-Vis, ultraviolet-visible; AAS, atomic absorption spectrometry; ELISA, enzyme-linked immunosorbent assay.

#### Ethics approval

Ethical approval was obtained from the Public Health Foundation of India’s Institutional Ethics Committee. Adult male respondents participated in the study. The female participants were mostly married and were mothers. Although there was a possibility of inclusion of younger women (less than 18 years) who were married at an early age and had children, we did not seek parental consent because we did not consider them as dependent adolescents in the group. Written informed consent was obtained from all participants who were literate. Third-party witnessed verbal consents were obtained from illiterate participants.

## Results

The present study was conducted in the villages of Bariyarpur, Kadampur, Tilabad, and Mahuatand of the Sunderpahari block of the Godda district of Jharkhand, India.

The outcome of our study related to indigenous knowledge and nutritive values of various traditional food items consumed by the Santhal tribal community is described below:

### Food consumption of Santhal community

Rice was the staple food for the community. The community consumed hybrid rice (varieties like Swarna, Pan patta [pan 819], and Chhabbis number), the seeds for which they bought from the local market. The participants preferred the taste, texture, and smell of traditional rice varieties such as jadhan, bahiyad, and bad but most of them had stopped cultivating it. Some families continued to store the seeds and grew them in smaller plots. These varieties were not for everyday household consumption but were only consumed on special occasions. During the pairwise ranking exercise, hybrid rice (Swarna) emerged as the most important cereal crop not because of taste but because it was readily available and affordable. Although the price of traditional rice varieties when sold were reported to be higher in the market, the yields were quite low. The hybrid rice was consumed mostly with green leafy vegetables. Pulses were consumed but not on a daily basis. The respondents reported consuming fleshy foods, which included wild meat, various birds, rodents, molluscs, and fish. Male members of the community still reported hunting for smaller animals in the surrounding forests but larger game was not hunted because it is prohibited by the government. Consumption of roots and tubers both cultivated and from the wild and fruits, especially wild fruits, was also reported. The availability and consumption of a large variety of GLVs were reported in all discussions. Many of these GLVs were also sundried and preserved for use at other times of the year. The *bari* or kitchen gardens provided a ready supply of GLVs and vegetables such as papaya, pumpkin, beans, gourds, lady finger, tomato, onion, garlic, brinjal, jackfruit, etc. Some households that had vegetables in excess of family requirements reported selling them in the market. Some GLVs were procured from the cultivated lands where they grew as weeds, some GLVs were the new leaves of trees within the village, and some were collected from the forest. The discussions revealed that the local government created artificial ponds that could be rented by individuals who were interested in spawning fish. These individuals and their families consumed the fish and sold the surplus during the weekly markets. Sometimes a couple of households would add their money and rent the ponds as a group and shared the fish among themselves.

Based on the focus group discussion, a free listing of all of the food items that were considered as indigenous foods by the participants was prepared along with their edible parts. The details are provided in Table 3.

**Table 3.** Indigenous foods with edible parts and the source.

Name of the food item	English name	Part consumed	Accessed/grown
Jondra/desi makka	Maize	Grain	Market
Swarna-bad/bahiyad, sorob, chinabora, jadhan	Rice varieties	Grain	Field,market
Bhadai			
Kulthi, lahar	Pulse varieties	Seed	Market
Sutro, khesari, ghangra			Farm
Barbatti			Jungle
Seem/van murgi	Wild hen	Meat	Forest
Kulhai/khargosh	Rabbit	Meat	Forest
Guddu/moosa	Field rat	Meat	Field
Ghonga	Snail	Meat	Farm
Bir sukri	Wild pig	Meat	Forest
Sigga/tood	Squirrel	Meat	Forest
Saahi/gheenk	Porcupine	Meat	Forest
Mahlah	Cat-like animal		Forest
Rundra/chota siyar/van bilaar	Cat-like animal	Meat	Forest
Panduk/chidiya	Sparrow	Meat	Forest
Jhingi	NA	Meat	Forest
Parwa/kabootar	Pigeon	Meat	Market
makhar	*Koel*	Meat	Forest
Puthi, ich, chatgoi	Varieties of fish	Meat	Pond
Lindra, doodi, gadai, mangri, sising, bhambui, golden, litoor, chepre, gogli			
Jhinuk	Mussel	Meat	Pond
Taaro, tarop/piyar, mirle/kataar, miraal, tiril/kendu, luya, podo bili, khudi rama	NA	Fruit	Forest
Koot			Kitchen garden, field
Ul/desi aam	Mango	Fruit	
Janum/ber	Zizyphus	Fruit	
Bhelua	Marking nut	Fruit	Forest
Amda, barhu/kusum ka phal, dahu	Ambada, kusum fruit	Fruit	Field, forest
Kwindi bili	Mahua	Fruit	Forest
Taad ka phal	Palm fruit	Fruit	Field
Sin-arak/kondra-arak, munga-arak, suya-arak, taaben-arak, jalibi (sweet tamarind), susni-arak, kantha-arak, garundi-arak, chauri-arak, dhurup-arak, kana-arak, lapong-arak, geetil-arak, Sirgiti-arak/siliary	Varieties of green leafy vegetables	Leaves	Weed, kitchen garden
Pindiya-arak, teeri reeti, thampt-arak, ohoic-arak			Weed, field
Hesak-arak, matha-arak			Weed, forest
But-arak, kaddu-arak			Kitchen garden
Allu-arak, saru-arak, pindiya-arak			Field
Daari Gandhari			Market
Sem (3 varieties), bada ghangra	Field beans	Vegetable	Field
Kukri	Sweet variety of bitter gourd		Kitchen garden, market
Bir karela	Small variety of bitter gourd		Forest, field
Pindra			Field
Pindarkoo			Field
Hoterba	Jute	Stem	Forest, field
Kapu		Tuber	Forest
Busu, butu, machi, turmal, damandi, putka, semhu, jangali chhaati	Varieties of mushroom	Mushroom	Forest, field
Kwindi tel	Mahua oil	Oil (from seed)	Market
Hadiya	Rice alcohol	Fermented rice preparation	Home, market
Taadi	Palm alcohol	Prepared from palm	Home, market
Mahua	Mahua alcohol	Prepared from mahua	Market
Khajur tadi	Date alcohol	Prepared from fresh dates	Home, market

### Outcome of pairwise ranking

**Figure 1.**
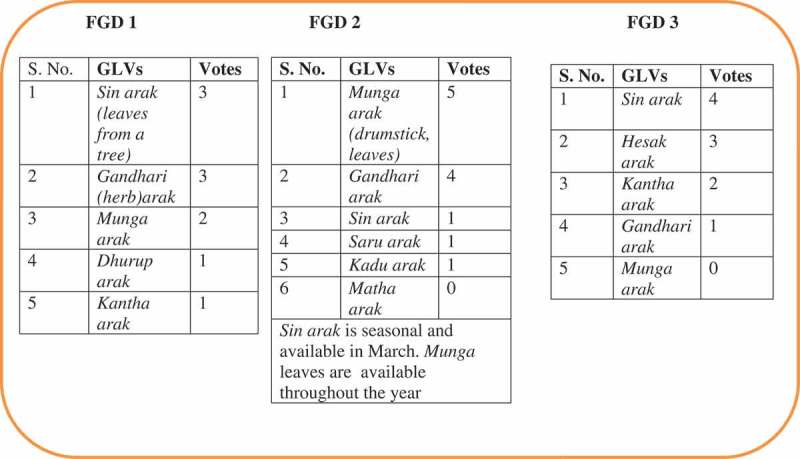
Outcome of pairwise ranking.

The commonly consumed indigenous foods under each food group were identified using the exercise of pairwise ranking; one such example for preferred GLVs identified during different FGDs is demonstrated in Figure 1.

In the study villages, among cereals, Jadhan, Bahiyad, and Bad, followed by Lohna, were the most common indigenous variety of rice cultivated and consumed along with the hybrid varieties of rice, which were either the most preferred or equally preferred to indigenous varieties. Among the GLVs, sin-arak, gandhari-arak, mung-arak, susni-arak, matha-arak, kantha-arak, hesak-arak, dhurup-arak, and saru-arak (*arak* means green leafy vegetable) were the most preferred and consumed varieties. Out of these, sin-arak, a seasonal variety of GLV, was the most preferred one, followed by munga-arak, which was available throughout the year. In case of vegetables and roots and tubers, no indigenous variety was commonly consumed except for sem (a kind of bean). Kulthi dal was the most preferred indigenous variety of pulse.

### Taxonomic classification of indigenous foods

**Figure 2.**
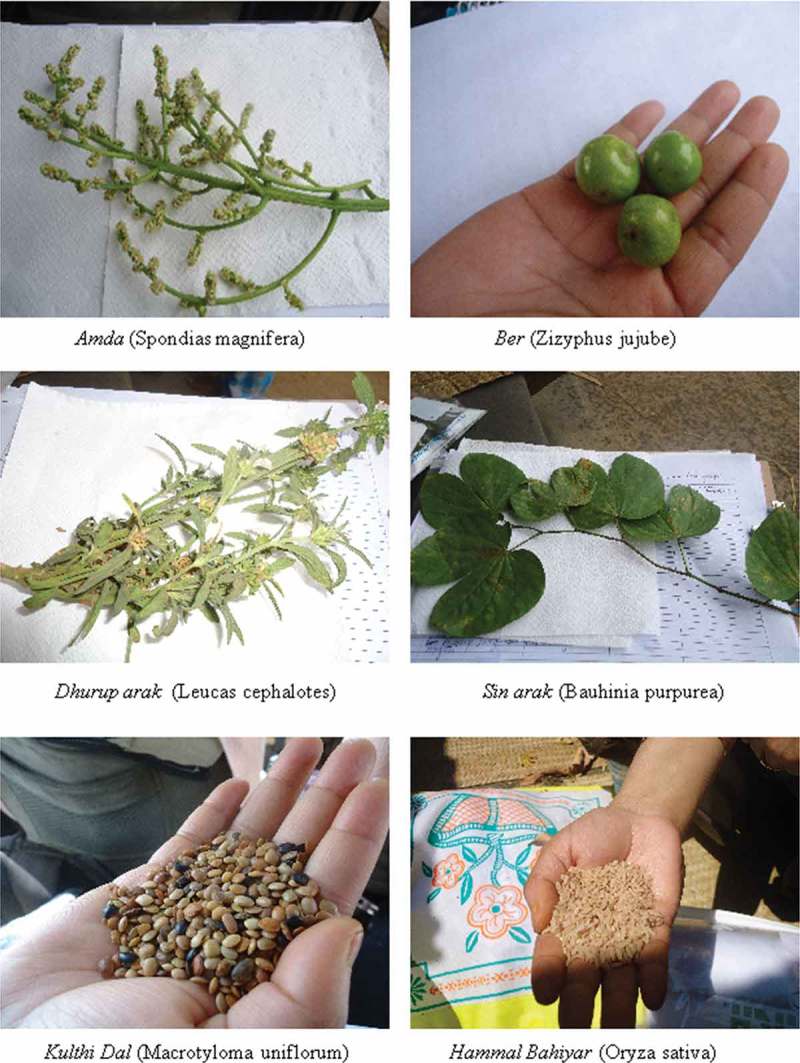
Indigenous foods of Santhal tribal community of Jharkhand.

**Figure 3.**
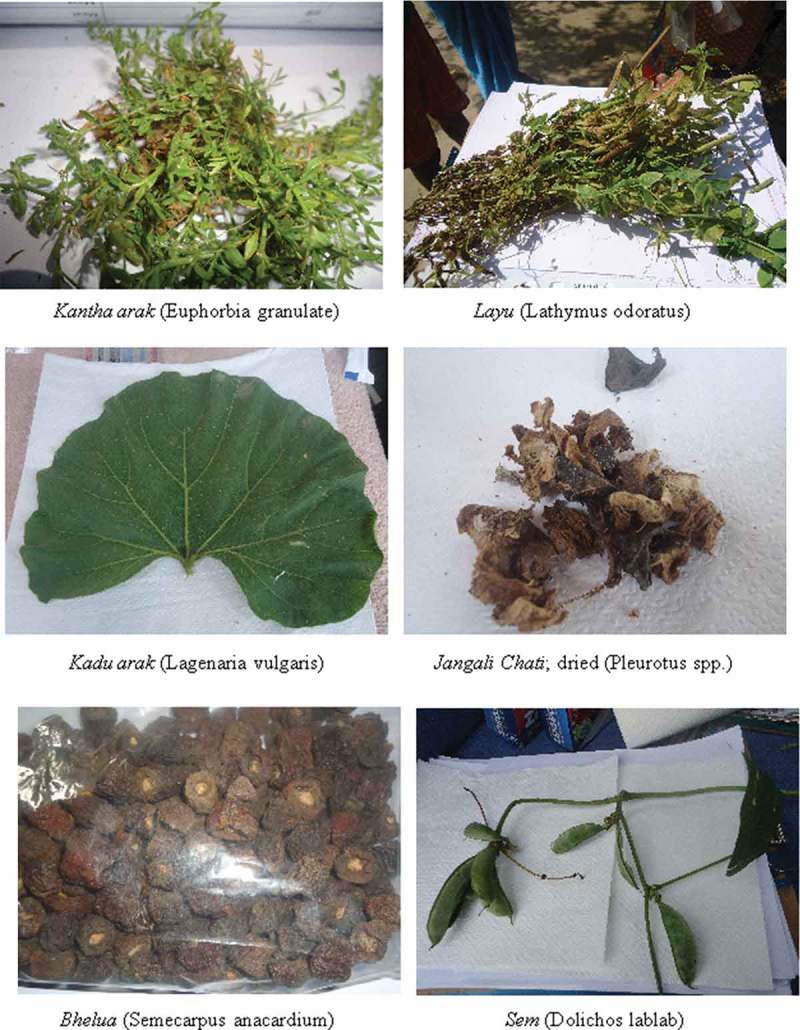
Indigenous foods of Santhal tribal community of Jharkhand.

A literature search was done in order to identify these foods based on their local names. For 25 foods, taxonomic classification was available based on their common names in the Indian food composition tables^29^ and other secondary data sources. For foods for which data from the literature were not available, samples based on availability (*n* = 26) were collected and sent for identification and taxonomic classification to the Birsa Agricultural University (Botany Department). The scientific names were also corroborated from other literature sources including Indian food composition tables (Table 4). Some representative photographs of these foods taken by the research team are provided in Figure 2 and Figure 3.

**Table 4.** List of indigenous foods collected and sent for classification along with verification from other sources of literature.^a^

S. No.	Local name of sample collected	Identification done by the botanist		Verification from other sources
		Genus	Species	
1	Hammal bahiyar (rice)	*Oryza*	*sativa*	*Oryza sativa*^28^
2	Swarna (rice)	*Oryza*	*sativa*	*Oryza sativa*^28^
3	Layu (dried) (millet)	*Lathymus*	*odoratus*	Layo, *Panicum antidotale* Retz.^32^
4	Sutro (pulse)	*Phaseolus*	*calcaratus*	*Phaseolus calcaratus* (common name, sutri)^28^
5	Ghangra dal (pulse)	*—*	*—*	*Dolichos iat* Jang, Ling.^34^
6	Gandhari-arak (GLV)	*Paederia*	*foetida*	*Amaranthus spinosus*^28^
7	Dhurup-arak (GLV)	*Leucas*	*cephalotes*	*Leucas cephalotes* Spreng. (Labiatae)^32^
8	Sin-arak/kondra (GLV)	*Bauhinia*	*purpurea*	*Bauhinia purpurea* (common name, kohar/konar sag)^28^
9	Jalibi (GLV)	*Pithecellobium*	*dulce*	—
10	Lotni (GLV)	*Brassica*	*juneca*	—
11	Kantha-arak (GLV)	*Euphorbia*	*granulata*	*Euphorbia granulata* Forsk. (Euphorbiaceae)^14^
12	Teeri reeti (GLV)	*Vicia*	*hirsuta*	Mentioned as pulse in Nollet and Toldra^34^
13	Garundi-arak (GLV)	Alternanthera	sessilis	*Alternanthera sessilis* R. Br. (Amaranthaceae)^14^
				*Alternathera sessilis* (common name, Ponnanganni)^28^
14	Taaben-arak (GLV)	*Portulaca*	*oleracea*	—
15	Susni saag (GLV)	*Marsilea*	*minuta*	*Marsilea minuta* Linn. (Marsiliaceae)^14^
16	Hesa-arak (GLV)	*—*	*—*	*Ficus religiosa*^28^
17	Chauri-arak (GLV)	*Scoparia*	*dulcis*	—
18	Lapong-arak (GLV)	*Aerua*	*lanata*	*Aerua lanata* Juss. Ex Schult (Amaranthaceae)^14^
19	Kadu-arak (GLV)	*Lagenaria*	*vulgaris*	*Lagenaria vulgaris*^28^
20	Layu (GLV)	*Lathymus*	*odoratus*	Layo, *Panicum antidotale* Retz.^32^
21	Ohoic-arak (GLV)	*Boerhaavia*	*diffusa*	*Boerhaavia diffusa* Linn.^14^
22	Lahsun saag (GLV)	*Allium*	*sativum*	*Allium sativum*^28^
23	Bada ghangra (vegetable)	*—*	*—*	*Dolichos iat* Jang, Ling^34^
24	Kapu (tuber)	*Dioscorea*	*bulbifera*	—
25	Jangali chati (mushroom)	*Pleurotus*	spp.	—
26	Bhelua (fruit)	*Semecarpus*	*anacardium*	*Semecarpus anacardium* (common name, marking nut)^28^

^a^GLV indicates .

### Nutritive value of indigenous foods

In addition to the food items documented in the Indian foods composition tables based on their common names, the nutritive values were available for 7 foods in the Indian food composition tables from among those classified by the botanists’ team at Birsa University. The nutritive values of both of these groups—that is, 7 foods along with foods identified earlier (whose nutritive values were available in Indian food composition tables, *n* = 32)—were compiled and studied. For the rest, food items were procured as per availability from the locality (*n* = 13), including 3 samples of indigenous alcohol, and sent for nutrient analysis to an NABL-certified laboratory in New Delhi. Table 5 provides the nutritive values of these newly analyzed foods.

**Table 5.** Nutritive values of foods analyzed in the laboratory.

S. No.	Parameter/food items	Moisture (%)	Energy (kcal/100 g)	Protein (g/100 g)	Total fat (g/100 g)	Total carbohydrate (g/100 g)	Sugar (g/100 g)		Ethanol (g/100 ml)	Total dietary fiber (g/100 g)	Vitamin A (beta carotene) (μg/100 g)	Vitamin B_1_ (mg/100 g)^a^	Vitamin B_2_ (mg/100 g)^a^	Vitamin B_3_ (mg/100 g)	Vitamin C as l-ascorbic acid (mg/100 g)	Calcium (mg/100 g)	Iron (mg/100 g)	Zinc (mg/100 g)	Sodium (mg/100 g)	Folic acid (μg/100 g)
1	Ohoic-arak (GLV)	82.6	53	3.4	ND	9.8	ND	—		6.1	16 010	ND	ND	ND	12	202	10.68	0.41	39.4	22.3
2	Lapong-arak (GLV)	81.6	56	4.6	ND	9.5	ND	—		5.9	21 760	ND	ND	7.03	19	322	22.06	0.65	10.4	40.5
3	Dhurup-arak (GLV)	80.1	67	5.7	ND	11.1	ND	—		6.7	18 460	ND	ND	ND	8	236	20.02	0.80	10.6	10.7
4	Lahsun saag (GLV)	90.1	34	3.1	ND	5.4	ND	—		4.9	5100	ND	ND	b	7	221	5.95	0.21	10.8	2.9
5	Kantha-arak (GLV)	83.6	46	3.5	ND	8.0	ND	—		7.1	11 680	3.07	ND	8.3	9	425	81.09	1.01	24.9	7.2
6	Hesa-arak (GLV)	65.6	121	2.9	ND	27.3	ND	—		22.3	8200	ND	ND	8.2	ND	295	2.77	0.80	7.5	3.9
7	Ghangra dal (pulse)	7.9	365	24.2	2.3	61.9	1.8	—		28.6	10	ND	ND	ND	5	91	6.07	3.83	4.25	17.89
8	Teeri reeti (pulse)	7.7	361	26.9	1.9	58.9	1.2	—		31.2	550	ND	ND	2.0	23	215	7.78	4.11	33.18	7.11
9	Jadhan (rice)	12.4	351	7.3	0.78	78.7	ND	—		4.9	ND	0.65	ND	5.33	ND	7	1.73	0.97	17.3	5.5
10	Bada ghangra (vegetable)	87.1	49	3.7	ND	8.5	ND	—		4.4	36	ND	ND	ND	9	41	0.95	0.61	1.86	7.0
11	Mahua (alcohol) (per 100 ml)^c^	80.8	134	ND	ND	ND	ND	19.11		—	ND	ND	ND	1.55	ND	4	ND	ND	2.1	ND
12	Hadiya (alcohol) (per 100 ml)^c^	96.5	20	ND	ND	1.6	ND	1.88		—	ND	ND	ND	1.59	ND	12	ND	ND	4.5	0.3
13	Khajur tadi (alcohol) (per 100 ml)^c^	92.0	45	ND	ND	2.7	ND	4.91		—	ND	ND	ND	ND	ND	7	ND	ND	3.3	0.26

GLV indicates green leafy vegetables; ND, not determined.

^a^The lab used a limit of detection of 0.1 mg/100 g for vitamins B1 and B2. These values are indicative of rich source of these vitamins. Hence, for most of the foods identified the value for these vitamins were not detected.

^b^Erroneous value.

^c^All parameters are per 100 ml for the alcohol varieties.

Many of the indigenous GLVs analyzed as a part of this study, namely, ohoic-arak (*Boerhaavia diffusa*), lapong-arak (*Aerua lanata*), and dhurup-arak (*Leucas cephalotes*), were found to be rich sources of calcium (range 202 to 322 mg/100 g of edible portion), iron (10 to 22.06 mg/100 g), and beta carotene (15,000 to 21,000 μg/100 g). Kantha-arak (*Euphorbia granulata*), another GLV, was found to be exceptionally high in iron (81.09 mg/100 g). One of the indigenous pulses, namely, teeri reeti (*Vicia hirsuta*), was found to be a good source of calcium (215.25 mg/100 g). Many indigenous foods for which nutritive values were available in the Indian food composition tables, namely, sin-arak (*Bauhinia purpurea*), garundi-arak (*Alternanthera sessilis*), gandhari-arak (*Amaranthus spinosus*), and matha-arak (*Antidesma diandrum*), have high levels of calcium (300 to 1717 mg/100 g); iron in gandhari-arak is reported as 22.9 mg/100 g, and beta carotene in garundi-arak and gandhari-arak is reported in the range of 1926–3564 μg/100 g. The vitamin C content of munga-arak is reported as 200 mg/100 g. Among lentils, kulthi dal (*Dolichos biflorus*) and sutro dal (*Phaseolus calcaratus*) are reported to be rich in calcium (200–300 mg/100 g). Fleshy foods like snail and freshwater mussel consumed by the community are rich sources of protein and calcium (592–870 mg/100 g). The majority of indigenous foods identified in the present study were found to be rich sources of calcium, iron, and beta carotene.

## Discussion

The search for novel, locally available, high-quality, inexpensive foods has continued to be promulgated as an important strategy for meeting nutritional requirements and addressing hidden hunger within a community. Maximizing the utilization of indigenous foods can be an important and sustainable dietary diversification strategy for addressing the nutritional needs of an indigenous population. In the present study, a total of 103 types of indigenous foods were identified (Table 1). These included a total of 25 indigenous varieties of GLVs, 6 varieties of pulses, 1 variety of tuber, 7 varieties of vegetable, 17 varieties of fruits, 8 varieties of mushroom, 7 varieties of cereals, 13 varieties of fleshy food items, 13 varieties of fish (plus mussel), and 1 variety of oil. Three varieties of alcohol brewed from locally available plant sources were also identified during the study.^44^

Analysis of nutritive values of the identified indigenous foods showed high levels of micronutrients like calcium, iron, beta carotene, and folate in many of them. A very high level of iron in kantha-arak (*Euphorbia granulata*) as analyzed in the present study has also been reported by Parvez et al.^45^ Studies have documented that indigenous varieties of fruits in communities with normal consumption patterns potentially contributed to fulfilling dietary recommendations of vitamin A.^46^ ^–^ ^51^ In addition, Ogle et al^52^ found that the daily intake of some naturally occurring vegetables can potentially contribute to fulfill up to 30% and 40% of the recommended allowances of vitamin A and calcium, respectively. A study by Singh and Garg^53^ showed that daily intake of a spice mix can contribute 5%–7% of the recommended daily allowances of some micronutrients (ie, chromium, iron, manganese, zinc, copper, phosphorous, and selenium). These studies strongly support the premise that the indigenous foods identified in the present study, if optimally consumed, can contribute to nutritional security and may address hidden hunger in the Santhal community. The consumption of mostly hybrid varieties of rice observed in the study villages is a cause for concern. This loss of crop diversity may lead to compromised nutrient intake because traditional rice varieties have more fiber and better nutrient composition than high-yield hybrid varieties. In some areas of Jharkhand, specific rice varieties are even used for medicinal purposes and given to lactating mothers or those suffering from dysentery.^54^

The results of our pairwise ranking clearly showed a strong preference for micronutrient-dense indigenous GLVs as part of the daily diet. Daily consumption of local foods is imperative for the food and nutrition security of people living in traditional societies and rural areas.^55^ Further, many of these GLVs were either procured from the wild or grew as weeds in cultivated and noncultivated lands. The local population was also aware of the manifold ways of utilizing these for regular consumption as well as methods of preservation for use during the seasons of the year when they are not available. Additionally, these foods can prove to be an important strategy to complement the routine iron, folate, and calcium supplementation interventions for improving maternal and child micronutrient status in these communities. The neglected and underutilized food resources that are present in indigenous food environments constitute the bedrock of the diversity in traditional and indigenous food systems of developing country communities and would be important in addressing challenges specific to indigenous groups. Studies have indicated a high prevalence of both macro- and micronutrient undernutrition in the Santhal community.^17^ ^–^ ^20^ This exists amidst a rich knowledge of traditional foods that have potential to contribute to micronutrient intake. Knowledge of the edibility of a wide variety of indigenous flora and fauna exists in the community. What is perhaps missing is the value associated with these foods in terms of their nutritional quality, which could be leading to suboptimal intakes. Thus, there is a need to create awareness about the nutritional quality of these indigenous foods and effectively package the message with promotion of indigenous foods through nutrition education and advocacy. Thus, continuous and sustainable use of indigenous and wild foods can be a cost-effective strategy to address nutritional security and lead to sustainable ecosystem health and nutrition for the Santhal tribal community of Jharkhand.

### Limitations

The study was an exploratory work where hitherto undocumented indigenous foods in the Santhal tribal community were listed and analyzed. However, due to logistic reasons including difficult terrain and sample transfer, only a limited number of food items could be analyzed. We believe that there is immense scope for building on this study to expand and consolidate the existing inventory of indigenous foods in the study community.

The laboratory work was outsourced to an NABL-certified lab. The researchers accepted the information provided about the standard procedures and methodology adopted by the laboratory.

## Conclusion

The indigenous foods identified in the study were found to be rich sources of micronutrients. These are foods that are adapted to the local agro-ecosystem and do not need any special inputs for their cultivation and sustainability. A substantial contribution to the nutrition security and nutritional status of this indigenous community could be made by promoting the consumption of indigenous foods through creation of an enabling environment for enhancing awareness about their nutritional benefits. Transferring knowledge of these indigenous foods along with their nutritive values to future generations would also facilitate their continued use. The present study may thus pave the path toward further investigations into quantitative consumption estimates of these foods by the community. This would provide information about their contribution to daily micronutrient intake and their potential for alleviating common nutritional deficiencies.

## Authors’ contributions

SGJ and AS conceived and designed the study with overall supervision from GG. MM and PK developed the qualitative tools and collected and analyzed the qualitative data. SGJ, PK, and AS supervised the collection, identification, and nutrient analysis of food samples. SGJ prepared the first draft of the article. GG, MM, and AS commented on drafts of the article. All authors contributed to critique and modification of the article and read and approved the final version. SGJ had final responsibility for the decision to submit for publication.
